# Understanding Breast Cancer (BRCA) Mutations Through TikTok

**DOI:** 10.7759/cureus.81883

**Published:** 2025-04-08

**Authors:** Tamara Lalovic, Alexandar Lalovic, Priyanka Raju

**Affiliations:** 1 Obstetrics and Gynecology, Tower Health Reading Hospital, West Reading, USA; 2 Obstetrics and Gynecology, Liberty University College of Osteopathic Medicine, Lynchburg, USA

**Keywords:** brca 1/2, brca gene mutation, healthcare social media, health-related social media, social media platform

## Abstract

TikTok is one of the biggest social media platforms where people look for support, seek advice, and educate themselves through videos created by other users. In today's world, people frequently turn to the internet for easy access to health and medical information; it is no surprise that TikTok has helped facilitate the creation of online support groups for those who are overcoming various illnesses and diseases. Breast cancer (BRCA) is worldwide the most common cancer in women, and while BRCA discussions on TikTok have been researched previously, discussions surrounding the BRCA susceptibility gene mutations have not. This study aims to explore content on TikTok related to BRCA mutations and assess its impact on public awareness and physician involvement. One hundred videos were watched on TikTok after searching “BRCA”, BRCA1”, or “BRCA2”. These videos were categorized into groups based on content creator and type of content. The content included discussion regarding preventative measures with BRCA mutations, BRCA and BRCA mutation, ovarian cancer and BRCA mutation, and various treatment options. The videos were also evaluated based on the number of views each video received. This study found that healthcare workers participated in BRCA-related TikTok videos at only 2.3% of all evaluated videos; 97.4% were created by laypersons. Of those videos created by non-medical personnel, 17.3% were educational. The majority (49.3%) were centered around preventative measures and treatments undergone after a BRCA mutation diagnosis. The treatment modality most discussed was double mastectomy at 68%, with 41.3% of those videos being preventative double mastectomies without a current cancer diagnosis. The video with the most views was with regard to motherhood and BRCA prevention at 2.3 million views; 10.4% of the videos discussed difficulties with BRCA in motherhood. Overall, this study highlights the importance of physician involvement in social media platforms. It also showcases how medical providers can use TikTok to better understand patients' needs and discussions outside of the office with regard to their diagnosis.

## Introduction

Difficult conditions, such as breast cancer (BRCA) or a diagnosis of a BRCA mutation, can lead to significant isolation. TikTok has taken over as one of the biggest platforms for people to not only entertain themselves but also look for support, ask for advice, and educate themselves. Watching videos of others undergoing similar diseases and conditions helps create a universe of online support. This exhibits how popular online peer-to-peer communities have become. As providers, it is important to gain insight into public and patient perception regarding their treatment when they leave the office. Providers can also use these platforms themselves to provide educational information to these communities away from the hospital.

In 2023, TikTok was the highest-grossing mobile application, based on data obtained from both the App Store and the Google Play Store. At 1.5 billion monthly active users, it is the fifth most popular social media platform in the world [1]. A study assessing how the internet affects patients’ experience of cancer showed that cancer patients in particular use the internet for a wide range of support as well as to gain knowledge [2]. This support and information might not be met through everyday conventional healthcare means. Therefore, understanding how the internet can enhance patient education and provide patient support is extremely valuable [2].

BRCA is the most common cancer in women [3]. There are more than 2.3 million cases of BRCA diagnosed every single year [4]. Mutations in BRCA genes have been a known significant risk factor for breast and ovarian cancers [5]. In women with a BRCA1 mutation, the life-long risk of developing BRCA is 85%, and that of ovarian cancer is 50%. For a woman with BRCA2 gene positivity, the risk of breast and ovarian cancer is 45% and 30%, respectively [5]. These are very staggering percentages and showcase the immense risk these women have. In 15-20% of families with BRCA, BRCA1 gene mutations were found. In families with breast and ovarian cancer, this number was up to 50% [5]. These numbers show how many families are affected by BRCA in addition to the number of women being diagnosed with the deadly disease. It is no wonder BRCA awareness videos have become exceedingly more popular, especially in a world where people look to the internet for medical facts and guidance. The CDC posted that, from July to December of 2022, 58.5% of adults in the United States used the internet to look for health or medical information [6]. Providers should also look to the internet to both learn more about what their patients are discussing with regard to their disease outside of the office, as well as use it as a means of providing and educating with correct information.

## Materials and methods

In order to execute this project, TikTok was downloaded, and a new account was created. Creation of a new account ensured that previous videos, which were watched on TikTok prior to this study, would not alter the algorithm for which videos would be presented. The TikTok search engine was used, and “BRCA”, “BRCA1”, and “BRCA2” were searched individually. "Breast Cancer" was not used in the search engine because the preliminary results of videos watched under that search showed many videos that were not related to cancers with BRCA gene mutations. Previous studies evaluated BRCA-related content on TikTok; however, none evaluated BRCA-specific content. A filter was applied on the app, allowing the videos to be sorted by their individual “like count” after searching the above keywords. This was done by searching for one of the three keywords above and then clicking on the three dots by the search engine. Filters pop up, and the videos can be sorted by relevance, like count, and date posted. The video category was also set to “unwatched” to ensure that previous videos would not pop up more than once; this prevented videos from being used twice in this study. This was implemented in a similar manner by going to the filers; the video category had an option of "all", "unwatched", "watched", "liked", and "people you follow". "Unwatched" was clicked, and the setting was applied. The TikTok videos were viewed in their entirety. The first inclusion factor that allowed videos to be included in this study was if they directly discussed cancer associated with BRCA or BRCA mutations. There was a total of 100 videos originally analyzed for this study, after incorporating only the videos that specifically mentioned BRCA or a BRCA mutation. In addition to that inclusion factor, videos were only kept if they had more than 1,000 views. Only including views with more than 1,000 views caused 23 videos of the 100 to be excluded, which left 77 videos. This was important as it showed what content was being viewed at a significant amount. An Excel sheet was used to categorize and classify the videos. On the Excel sheet, the videos were divided into whether they were watched when “BRCA” was put in the search engine, “BRCA1” was searched, or “BRCA2”. The number of views each video received was documented. From there, the videos were defined by the content that was in the video. The videos were described as “personal” or "informative”. They were classified as “personal” if they were not created by a medical professional and if they included a personal discussion of a diagnosis or a family member’s diagnosis of a positive BRCA mutation or BRCA-related BRCA. They were described as “informative” if they were created by medical professionals who were educating viewers on BRCA mutations or BRCA-related BRCA in general. In order to be "informative," the creator had to explicitly mention being a medical professional. This included a physician or advanced practice provider such as a nurse practitioner or physician assistant. Videos included in the “personal” category were further subclassified into videos that were “personal informative.” Videos were included if content creators tried to integrate BRCA-related teaching moments into their discussion of personal experiences. "Personal motherhood” was another subgroup; videos were included if the discussion included difficulty with diagnosis specifically related to being a mom. All the videos were then described and grouped into whether their content was related to BRCA-associated breast cancer, BRCA-related ovarian cancer, or preventative treatment after a BRCA gene mutation was identified without a cancer diagnosis. The last subclassification for all videos in this study described what specific treatments were discussed for BRCA-related cancers/preventative treatments: double mastectomies, lumpectomies, total laparoscopic hysterectomies, or chemotherapy. Tables 1-4 show the grouping of the aforementioned with the number of videos included in each. Of note, in Table 3 regarding the treatments discussed, it is important to note that, in some videos, there were overlaps of the treatment regimens mentioned. For example, some videos discuss chemotherapy as well as a double mastectomy.

**Table 1:** Subdivision of all videos BRCA: Breast cancer

Subdivision of All Videos (N=77)	Number of Videos
BRCA	43
BRCA1	17
BRCA2	17

**Table 2:** Content in "personal" videos BRCA: Breast cancer

Content in "Personal" Videos (N=75)	Number of Videos
BRCA-Associated Breast Cancer	34
BRCA-Associated Ovarian Cancer	4
No Cancer/Preventative Treatment	37

**Table 3:** Treatments discussed in "personal" videos

Treatments Discussed in "Personal" Videos (N=75)	Number of Videos
Double Mastectomy	51
Lumpectomy	1
Double Mastectomy + Total Laparoscopic Hysterectomy	3
Chemotherapy	14
No Treatment Discussed	14

**Table 4:** Preventative treatment discussed in the "personal preventative" group

Preventative Treatment Discussed in "Personal Preventative" Group (N=37)	Number of Videos
Double Mastectomy	28
No Treatment	6
Double Mastectomy +Total Hysterectomy	3

When analyzing the data, the total number of videos included was 77 (N=77), as explained above. There were 75 videos (N=75) that were included in the “personal” category and 2 (N=2) that were included in the "informative category." Additionally, there were only 37 (N=37) videos included in the "personal preventative" category. Descriptive analyses were performed using charts and graphs with the data obtained in order to best understand the topics of conversation.

## Results

**Figure 1:**
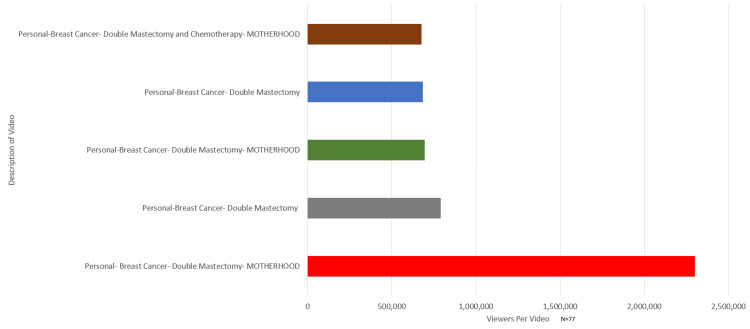
Top 5 most viewed BRCA-related TikTok videos BRCA: Breast cancer

A total of 100 TikTok videos were reviewed and analyzed. Of those 100, only 77 were used in the study as they met the aforementioned requirements (N=77). The videos with the largest number of viewers and their descriptions were documented, and the results are exhibited in Figure 1.

TikTok with the highest views received 2.3 million views. This TikTok was about motherhood with BRCA, discussing difficulties in receiving a BRCA-related cancer diagnosis as a mom. Three of the top 5 most viewed BRCA-related TikTok videos were regarding motherhood with BRCA. Motherhood with BRCA encompassed 10.4% (N=8) of the videos.

Only 2.6% (N=2) of the videos were disclosed by healthcare workers discussing BRCA1 and BRCA2 mutations/disease. The specific type of profession (nurse vs. physician assistant vs. physician) was not documented. 

**Figure 2:**
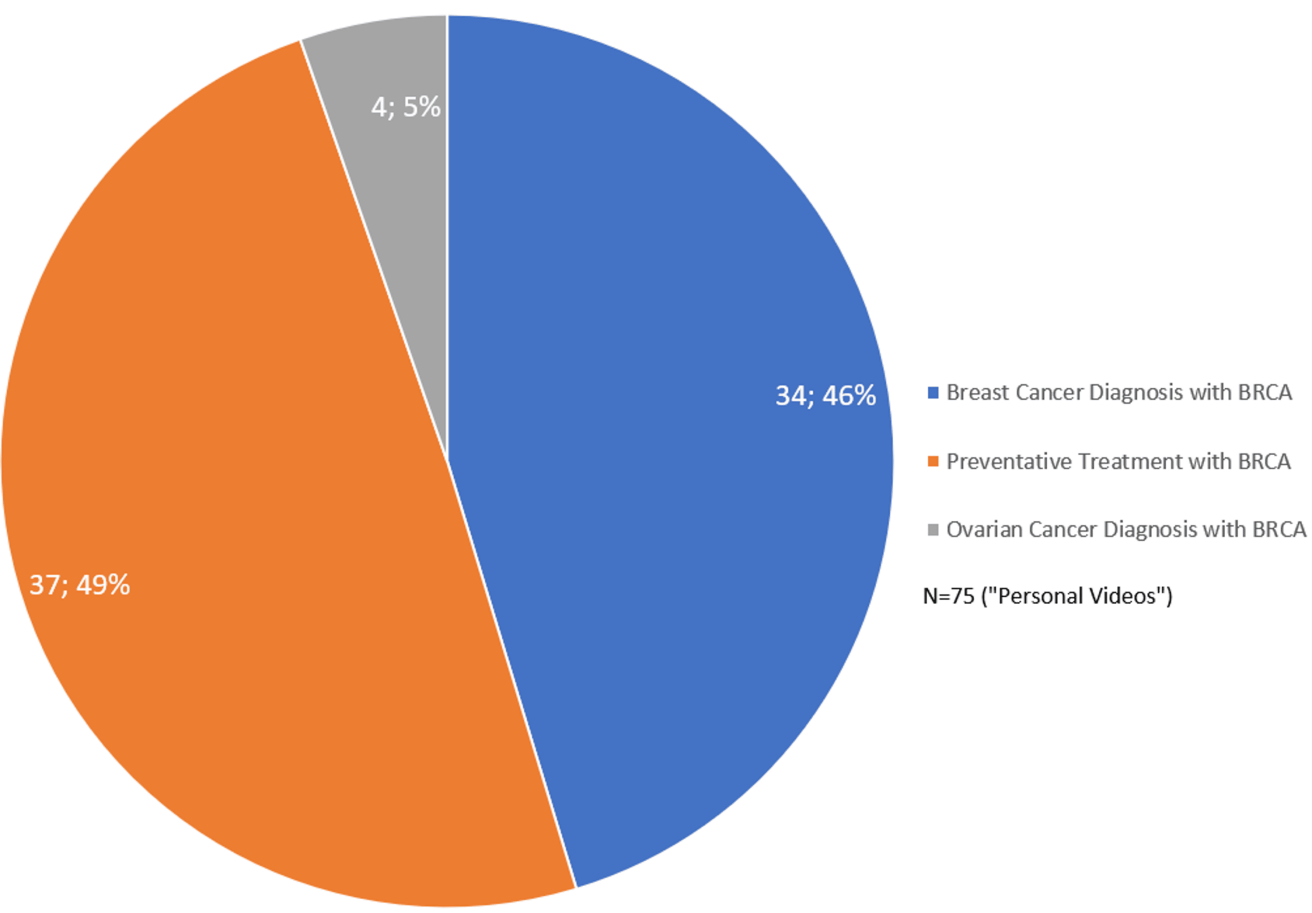
TikTok discussions by non-medical personnel

Additionally, 97.4% (N=75) of the videos were created by non-medical personnel. Of the videos created by non-medical personnel (N=75), only 17.3% (N=13) of those non-medical personnel videos were personally informative, with laypeople attempting to incorporate education in their videos. Figure 2 shows the distribution of videos created by nonmedical personnel. Specifically, 45.3% (N=34) of the non-medical videos were patients with a BRCA diagnosis associated with a BRCA mutation, and 49.3% (N=37) were centered around preventative measures being taken when diagnosed with a BRCA gene mutation. Only 5.3% (N=4) of the videos created by non-medical personnel discussed an ovarian cancer diagnosis with a BRCA mutation.

**Figure 3:**
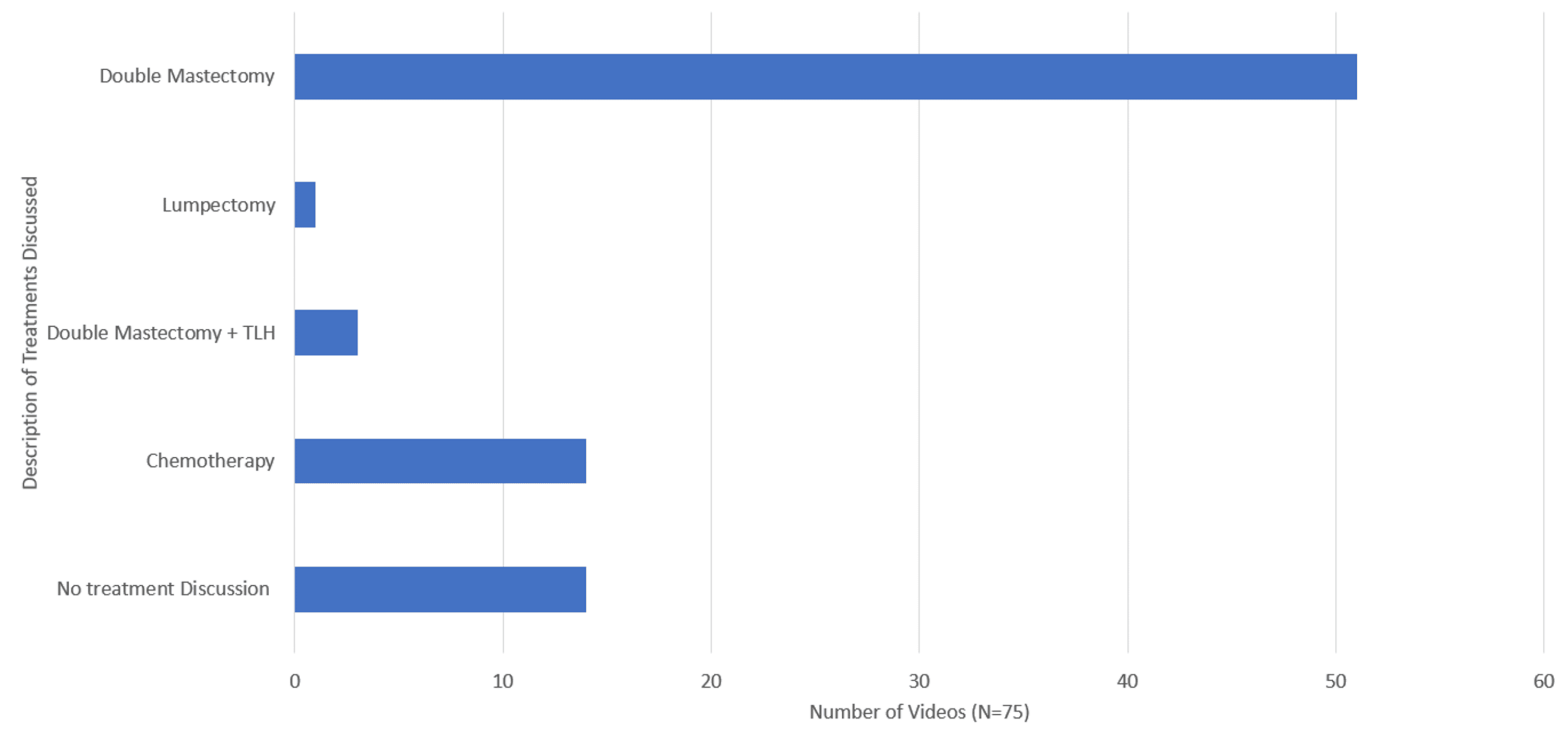
TikTok treatment discussion by non-medical personnel in the setting of BRCA gene mutation BRCA: Breast cancer

Of all the videos created by non-medical personnel (N=75), 18.7% (N=14) did not discuss any treatment modalities. Meanwhile, 68% (N=51) discussed double mastectomies, and of those, 41.3% (N=31) were preventative double mastectomies after a BRCA gene mutation was identified. Only 1.3% (N=1) discussed lumpectomy treatment. Chemotherapy was discussed in 18.7% (N=14) of non-medical personnel videos. Double mastectomy with total hysterectomy was discussed in 4.0% (N=3), 100% (N=3), which were done preventatively. Figure 3 shows the distribution of treatment discussion.

**Figure 4:**
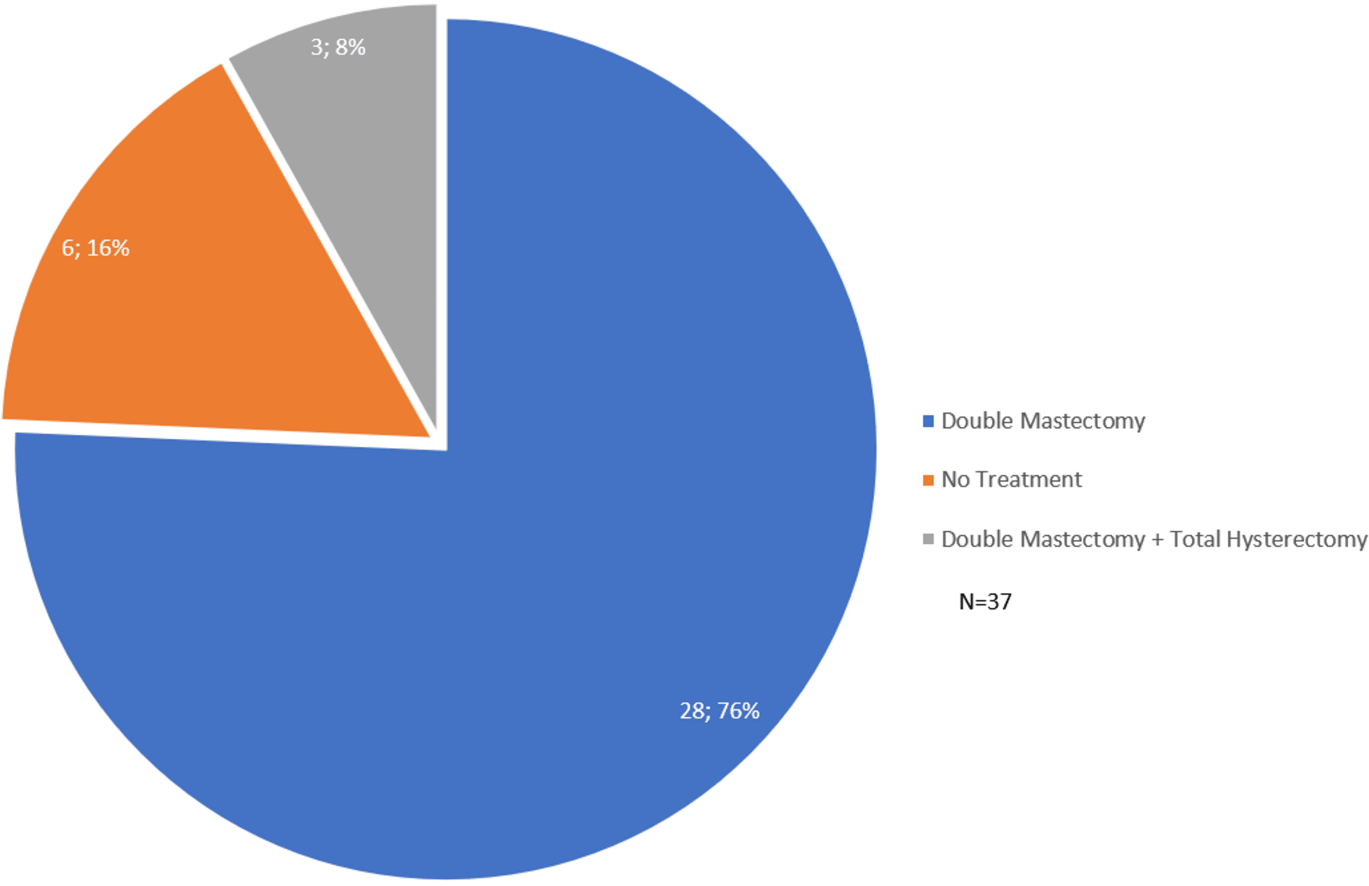
TikTok discussions regarding preventative treatments chosen after BRCA mutation diagnosis BRCA: Breast cancer

Figure 4 displays the discussions regarding preventative treatments. Out of all the discussions regarding preventative treatments (N=37), 75.7% (N=28) were regarding undergoing double mastectomies, 16.2% (N=6) were regarding no treatments being performed, and 8.1% (N=3) were regarding undergoing a double mastectomy with total hysterectomy.

## Discussion

These data show a lack of medical professional presence on TikTok regarding the topic of BRCA gene mutations. The vast majority of videos (97.4% of the reviewed videos) were created by non-medical personnel. This highlights a huge gap where physicians and advanced practice providers can fill with educational and supportive videos. This potentially opens space for TikTok to create a TikTok education platform, allowing providers to create accurate and informative videos. An integral aspect of patient care regarding patients with a diagnosis of a BRCA gene mutation is the ability to support patients outside of the office. Being able to support patients outside of the office on a BRCA diagnosis is very important to patient care. Interestingly, the majority of the videos created on TikTok were found to be non-medical personnel discussing preventative treatment options after their BRCA gene mutation was discovered. This can be correlated to patients’ interest in preventative treatment options. Of all preventative treatments, the most discussed on TikTok were double mastectomies at 75.7%. Physicians can potentially use this information to further educate others on the process of receiving a double mastectomy, as there is a strong presence of this topic on TikTok, or they can use the platform to highlight other preventative measures to further inform people on all of their various options. Similarly, double mastectomies were most talked about when adding in patients who also had a cancer diagnosis. Lumpectomies were very rarely talked about, at only 1.3%. As stated above, many people look to the internet for medical education and support; TikTok has stood out recently as a very prominent and popular social media platform. Previous studies have looked at overall BRCA discussions on TikTok. One study found that out of 100 TikTok videos viewed, the majority were filled with support and advocacy [7]. Similarly, a study focusing on understanding BRCA surgery on TikTok found that of 138 videos analyzed, the most common creator type was patients, at 77.3%. Physicians only created 10.3% of the videos. The study also found that storytelling was the topic of the video in 57%, while educational videos only encompassed 20% of all TikTok videos [8]. Qu et al. [9] assessed the quality of videos found on TikTok and found that patient videos frequently centered on personal experiences, while medical professionals primarily spoke on the stages of BRCA. In addition, the study concluded that medical professionals created videos of higher quality when compared to non-medical personnel. The article encouraged medical professionals to increase their involvement on TikTok, as this encourages the spread of accurate, reliable, and high-quality content [9]. Increased physician presence on TikTok also decreases current established limitations of using social media to spread medical information. These limitations include undisclosed conflicts of interest, unchecked spread of misinformation, and difficulty identifying source credibility [10]. As these studies all show, there is room for improvement with regard to physician-led content on this very popular social media platform. Physician involvement can lead to overall feelings of support and trust in the videos being watched on TikTok. Similarly, TikTok can be used by physicians as a means of understanding what is important to patients and what they discuss outside of the office. For example, 10.4% of videos in this study discussed motherhood with a BRCA diagnosis; the most viewed video at 2.3 million views was about motherhood with a BRCA diagnosis. This highlights the importance of discussing how patients feel about their diagnosis while taking their children and motherhood into consideration. Physicians can utilize this to encourage discussion of parenthood and being a mom when counseling their patients after a BRCA mutation detection. Overall, TikTok and other social media platforms can be used to provide information to patients seeking educational and supportive videos and as a way to understand what patients value and discuss outside of the office.

The greatest limitations in this study include the small sample size. Unfortunately, there were not as many videos discussing BRCA mutations as there were videos highlighting various other topics on TikTok, such as BRCA. Another limitation includes the inability to filter out multiple videos created by a single user. It is uncertain how many of the videos were by similar users who use the platform to discuss BRCA. While we were able to prevent the same video from being seen twice, we were not able to prevent the same user from being included in this study more than once.

## Conclusions

In conclusion, this study highlights the need for medical provider involvement on social media platforms, such as TikTok. This is valuable so that medical providers can create accurate educational content and partake in these online peer-to-peer support groups. It is also important for medical personnel to use these platforms to better understand discussions being had outside of their office. Physicians will be able to better counsel their patients if they know what trending topics are being watched and discussed on this exceedingly popular application. Utilizing social media platforms appropriately can only better help physicians connect to their patients and provide excellent care both in and out of the office.
